# A Review of Environmental Contamination and Health Risk Assessment of Wastewater Use for Crop Irrigation with a Focus on Low and High-Income Countries

**DOI:** 10.3390/ijerph15050895

**Published:** 2018-05-01

**Authors:** Sana Khalid, Muhammad Shahid, Natasha, Irshad Bibi, Tania Sarwar, Ali Haidar Shah, Nabeel Khan Niazi

**Affiliations:** 1 Department of Environmental Sciences, COMSATS Institute of Information Technology, 61100 Vehari, Pakistan; sanakhalid@ciitvehari.edu.pk (S.K.); natasha564ag@gmail.com (N.); taniasarwar911@gmail.com (T.S.); dralienvironmentalist@gmail.com (A.H.S.); 2 Institute of Soil and Environmental Sciences, University of Agriculture Faisalabad, Faisalabad 38040, Pakistan; irshad.niazi81@gmail.com; 3 MARUM and Department of Geosciences, University of Bremen, D-28359 Bremen, Germany; 4 Southern Cross GeoScience, Southern Cross University, Lismore, NSW 2480, Australia

**Keywords:** wastewater, heavy metals, soil contamination, health risk, toxicity

## Abstract

Population densities and freshwater resources are not evenly distributed worldwide. This has forced farmers to use wastewater for the irrigation of food crops. This practice presents both positive and negative effects with respect to agricultural use, as well as in the context of environmental contamination and toxicology. Although wastewater is an important source of essential nutrients for plants, many environmental, sanitary, and health risks are also associated with the use of wastewater for crop irrigation due to the presence of toxic contaminants and microbes. This review highlights the harmful and beneficial impacts of wastewater irrigation on the physical, biological, and chemical properties of soil (pH, cations and anions, organic matter, microbial activity). We delineate the potentially toxic element (PTEs) build up in the soil and, as such, their transfer into plants and humans. The possible human health risks associated with the use of untreated wastewater for crop irrigation are also predicted and discussed. We compare the current condition of wastewater reuse in agriculture and the associated environmental and health issues between developing and developed countries. In addition, some integrated sustainable solutions and future perspectives are also proposed, keeping in view the regional and global context, as well as the grounded reality of wastewater use for crop production, sanitary and planning issues, remedial techniques, awareness among civil society, and the role of the government and the relevant stakeholders.

## 1. Introduction

Water is an essential component of life, but it is a susceptible and finite resource that has qualitative vulnerability and quantitative limitations. It is expected that 60% of the total world’s population may face the problem of a water shortage by the year of 2025 [1]. In the areas where fresh water is in short supply, wastewater is frequently used for crop irrigation. Although not legally permitted in most countries, the use of untreated wastewater for crop irrigation has been practiced in many countries worldwide due to the shortage of good quality water [2,3,4].

Wastewater production sources include different human activities, such as industrial, commercial, and domestic activities. Municipal wastewater is also sometimes distinguished into urban, rural, and agricultural areas/sources. With the rapid expansion of the population, cities, industries, and the domestic water supply, the quantity of wastewater production is increasing at the same proportion [5]. The average volume of wastewater generated daily by human activities depends on the availability of the water quantity in the house, the cultural level/type, the cost of the water, and the economic conditions [6].

Agriculture is the most common area in which untreated wastewater is reused. According to an estimate in 2004, approximately 20 million ha is irrigated with wastewater in fifty countries worldwide [7,8]. The use of wastewater for crop irrigation has further increased in recent years. The municipal wastewater demand corresponds to 11% of the water withdrawal, globally [9]. About 3% of the municipal wastewater demand is consumed and the remaining 8% is being discharged as wastewater; that is, 330 km^3^ of wastewater per year [9], which is potentially irrigating almost 40 million ha (approximately 8000 m^3^ per ha) [9] or 15% of all irrigated lands.

Wastewater usage for crop irrigation has certain advantages such as providing the essential nutrients and organic matter, saving water and nutrients, and reducing water contamination [2,10]. It has been reported that quite sufficient quantities of macronutrients (N, P, and K) are supplied to soil and plants via wastewater application [10]. Therefore, it is a great temptation for poor farmers to irrigate crops with wastewater as it can reduce the crop production cost [11], with the cost of crop production decreasing by 10%–20% when irrigated using wastewater.

**Figure 1:**
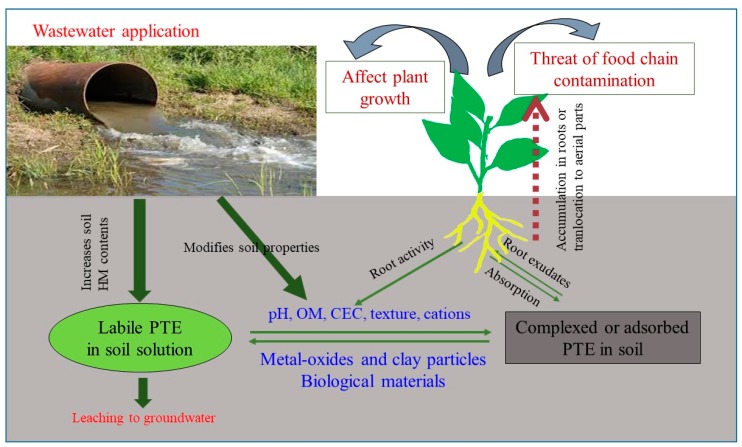
The possible environmental contamination by the use of wastewater; (PTE: potentially toxic elements).

Besides these benefits, a number of drawbacks are associated with the use of wastewater for crop irrigation [4,10,12]. Wastewater contains potentially toxic elements (PTEs) such as zinc, chromium, copper, cadmium, nickel, lead, mercury, and parasitic worms, which can induce severe risks to the human health and the environment [13,14,15]. The use of untreated wastewater for crop irrigation can also cause soil hardening and shallow groundwater contamination [16,17]. However, the main problem of wastewater crop irrigation is the presence of potentially toxic elements (PTEs) [4,18,19] (Figure 1). The build-up of PTEs in the soil and crops by wastewater irrigation results in soil contamination, and in turn, affects food safety [4,20,21].

The contamination of agricultural soils by wastewater application poses health risks due to the presence of PTEs which have long-term implications for the environmental and health [22,23]. Soil contamination with PTEs is the main route of their exposure to humans via food crop consumption, which could cause various health issues in humans if the PTE concentration exceeded the safe limit [24,25,26,27]. The long-term use of PTE-contaminated vegetables can cause the continuous build-up of toxic metals in the kidney and liver of humans, causing disorders in the physic-biochemical processes [28,29,30]. Studies regarding the health risk assessment after the consumption of PTE contaminated food/vegetables are being performed in developed countries. However, very little is explored in less developed countries [20,28,31].

However, there exists a considerable difference in the collection, treatment, and reuse of wastewater for crop irrigation between low and high-income countries [10]. This variation among the two groups can be due to several factors such as the availability of fresh water for crop irrigation, the availability of resources to treat wastewater, the awareness among the farming community about environmental and human health issues related to wastewater crop irrigation, and the implementation of laws of wastewater use in the agriculture sector [4,10,12]. Moreover, there are social, economic, and corporate issues that are also affecting the wastewater treatment and reuse for crop irrigation between low and high-income countries.

There exists abundant data regarding the generation of wastewater, its use for crop irrigation, and the associated environmental and health risks at the national and global level. However, there are contrasting opinions about the value of wastewater for the irrigation of crops. Some studies valued it as a source of essential crop nutrients and a management practice, while opponents claimed it an act of criminal negligence owing to its environmental and health issues [20,28,31]. A comprehensive review highlighting all these aspects at a national and international scenario can be of great use for researchers, scientists, and policymakers. Therefore, in this review, we highlight and compare the current scenario of wastewater production and use for crop irrigation and the associated environmental and health risks at the national and global level. Moreover, the review traces the history of wastewater use for crop irrigation and proposes some future perspectives and management strategies to minimize the risks associated with the use of wastewater for crop irrigation.

## 2. The Current Global Scenario of Wastewater Use for Crop Irrigation

Nowadays, under freshwater scarce conditions, it becomes almost mandatory for farmers to consider and use any sources of water, especially in many arid and semi-arid regions [12]. Consequently, crop irrigation using wastewater becomes a valid option, urged by the lack of feasible alternatives [10]. The agricultural sector uses a major portion of the total municipal water reuse [32], which accounts almost 70% of the total agricultural water consumption [33]. It has been reported that untreated or partially treated municipal/industrial wastewater is used for the irrigation of about >20 million ha of crop worldwide [2].

Nowadays, the use of wastewater for crop irrigation has gained considerable attention. With the rapid advancement, development, and wide acceptance/application of wastewater treatment technologies, the use of treated wastewater has increased in developed countries [34,35]. Globally, the use of wastewater for crop irrigation has increased about 10–29% per year in Europe, the United States, and China, and by up to 41% in Australia [35]. Table 1 presents the data about the total wastewater produced, collected, treated, and used for crop irrigation in different countries around the globe [9].

The global wastewater discharge reaches 400 billion m^3^/year, contaminating ~5500 billion m^3^ of water per year [2]. The nature and the quality of the wastewater used vary within and between the countries. It has been stated that 15 million m^3^/day of reclaimed water is reusing by approximately 44 countries for irrigation purposes [36]. Globally, the use of untreated wastewater for urban and peri-urban agriculture accounts for about 11% of all the irrigated croplands [37]. It is estimated that 10% of the global population consumes food produced from wastewater irrigation [38].

The practice of crop irrigation with wastewater is not well-regulated in low-income countries, and the environmental and economic issues are poorly understood [10,12]. Some poor countries use raw sewage for irrigation purpose, although its use is considered illegal [39]. In less developed and low-income countries such as Asia, Latin America, and Africa, wastewater is used for irrigation without any treatment [5,40]. However, on the other hand, in middle-income countries, wastewater is used after treatment [9].

Wastewater use for crop irrigation is practiced in several countries globally [41]. According to the estimation in fifty countries, wastewater is used to irrigate 20 million ha [42]. China is ranked the number 1 country in the world based on the total population and is also the top-ranked country by the volume of wastewater generated. About 68.5 billion tons of wastewater was discharged from municipal and industrial sources in 2012, which is comparable to the annual flow volume of the Yellow River [43]. Furthermore, the 1st National Pollutant Source Census Bulletin [44] estimated that about 108.16 billion cubic meters of wastewater (34.33 billion cubic meters from domestic sources and 73.83 billion cubic meters from industrial sources) were being generated per year in China.

It has been reported that the wastewater produced in China annually is used for irrigating an area of about 1.33 × 10^6^ ha [45]. Although China has adopted urban wastewater reuse programs, its development at the national level is slow. In India, about 38,354 million liters of wastewater is produced per day in major cities, while the treatment capacity is only 11,786 million liters per day. In India, approximately 2600 million m^3^ of untreated wastewater is used for crop production [19]. It has been estimated that about 73% of urban wastewater generated in India remains untreated (Development Finance Corporation) [46]. In Mexico, about 70,000 ha are irrigated with processed wastewater, while about 260,000 ha is irrigated with untreated wastewater [47]. Informal irrigation in Ghana involving wastewater diluted from streams and rivers occurs on 11,500 ha of an area larger than the reported extent of the formal irrigation in the country [48].

## 3. The Wastewater Use and Treatment in Low and High Income Countries

**Figure 2:**
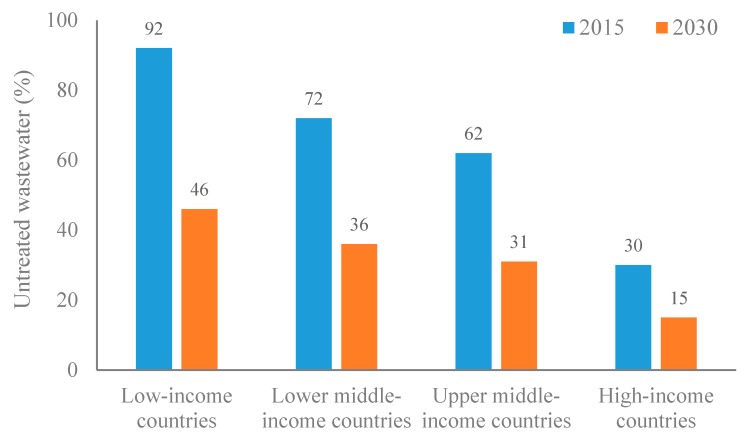
The percentage (%) of untreated wastewater discharged into the environment in low and high-income countries.

Globally, about 330 km^3^/year of municipal wastewater is generated, which can theoretically irrigate and fertilize crops grown on millions of hectares. However, the fate of this wastewater is very different in low and high-income countries. In the literature, the data on the fate (collection, treatment, and reuse) is scarce and scattered globally. Only a small portion of waste is currently treated and the share of the treated wastewater that is used for crop irrigation is significantly low. According to the Food and Agriculture Organization (FAO) [9], globally, about 60% of the total municipal wastewater generated is treated before its reuse. The data on the percent of untreated wastewater discharged into the environment is presented in Figure 2 [49]. It is evident from Figure 2 that the wastewater released into the environment, in the treated or untreated form, greatly varies between low and high-income countries. Currently, 92% and 30% of untreated wastewater is discharged into the environment, respectively, in low and high-income countries [9].

High-income countries have established wastewater treatment facilities. It has been reported that, globally, more than 80% of the facilities of wastewater treatment have been identified in developed countries such as the USA, Japan, and Europe. For example, in France, there are over 17,000 wastewater treatment plants and 15,000 water treatment plants. The sewerage network in France is nearly 800,000 km long. China has 3272 urban sewage treatment plants, which can treat about 140 million cubic meters of sewage per day (Ministry of Housing and Urban-Rural Development of the People’s Republic of China) [50]. The USA had 15,591 treatment facilities in 2000. In California, more than 75% of the processed wastewater is used for crop irrigation.

Low-income countries have very low or limited wastewater treatment facilities. For example, in east India, wastewater treatment plants neither exist nor function adequately [51]. In low-income countries, the wastewater released from industrial and municipal sources is used for crop irrigation directly, with no or very little dilution. In some cases, the wastewater is first released into water bodies where it undergoes dilution prior to its use for crop irrigation. In this way, the effect of wastewater irrigation on the soil, plants, and human health may vary when used with or without prior dilution.

In many low-income countries, the absence of technical and financial resources makes it very difficult to efficiently collect and treat wastewater. For example, approximately 30% of the total wastewater produced is directly used for the irrigation of 32,500 ha of crops in Pakistan [52], while 64% of the wastewater is directly discharged into water bodies without any pretreatment [4]. In India, only 24% of wastewater from the industrial and municipal sectors are treated [53].

The situation of wastewater collection and treatment is even worse in West African countries, where usually <10% of the wastewater produced is collected in sewage systems [54]. Firstly, the wastewater treatment facilities are totally missing in developing countries. In the cases where the wastewater treatment facilities are available, the sustainability of these facilities is a big issue in developing countries. In many cities in Asia and Africa, large centralized wastewater collection and treatment plants/systems have failed to be sustained and are not functional.

Consequently, the decentralized wastewater collection and treatment systems have been promoted in low-income countries as they are more flexible for long-term operation and financial sustainability [55]. For example, in Ghana, only 7 out of 44 smaller treatment plants are operational and, generally, none of them meet the designed effluent standards [54].

There exists limited data in the published literature about water disposal and treatment, as well as its use in the agricultural sector of developing countries compared to developed countries. In fact, in developing countries, there are no proper university-industry research linkages, which is one of the main reasons for the lack of problem-oriented research in these countries. The lack of scientific/research data and university-industry research linkages in these countries makes it difficult to establish policies, planning, and laws for environmentally friendly practices, especially in the agricultural sector.

Moreover, the economic and social factors, as well as the lack of awareness and knowledge of the environmental and health risks among the people of developing countries also hinder the adaptation and implementation of these environmentally friendly practices. Although there is a significant improvement regarding the problem-oriented research in the last decade, especially in the Indo-Pak sub-continent, many key steps are needed for the proper implementation of environmentally friendly practices in developing countries. The role of research institutes/universities and funding agencies, as well as government organizations, could play a key role in this regard. Poverty is also considered an important factor behind the use of wastewater for crop irrigation, especially in less-developed countries. Farmers with small agricultural land holdings prefer to use wastewater for irrigation despite the availability of fresh water. This is due to the low-cost of crop production by adopting this irrigation practice.

In some developing countries, the limited public awareness, the less commercial development of wastewater recycle/reuse, and the social setup are some of the other key challenges faced in the realm of wastewater use for the agricultural sector. Wastewater recycling/reuse seminars, commercial operation mechanisms, and fiscal support from the government can be highly effective in mitigating the environmental and health issues related to the use of wastewater in the agricultural sector.

The above-mentioned facts clearly show an alarming gap of environmentally-friendly practices and environmentally sustainable approaches regarding water treatment among the developed and developing countries, especially in the highly populated (and continual growing) areas of South and Southeast Asia. There is a need at the global level to do more and shrink down this lacuna. In this regard, the role of the environmental and health organizations working at the national and international level is critically important.

## 4. Potential Impacts of Wastewater Irrigation on Crops

**Figure 3:**
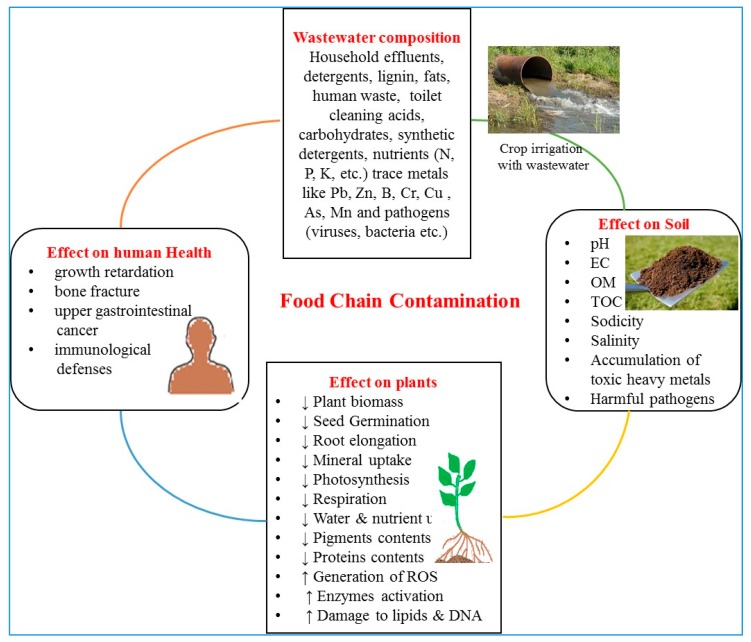
The possible food chain contamination by wastewater crop irrigation.

The use of wastewater for crop irrigation in the agricultural sector has the potential for both negative and positive effects (Table 2) on the soil quality/productivity, crop production, and human health [4,12] (Figure 3). Wastewater may contain unwanted pathogens and chemical constituents that pose health and environmental risks. The negative effects of crop irrigation with wastewater are primarily due to the presence of high total suspended and dissolved solids, high nutrient contents, and PTEs [3,4,16,56]. Wastewater may contain high concentrations of salts which can affect the soil quality and productivity by accumulating in the root zone. The prolonged use of saline and sodium-rich wastewater can deteriorate the soil structure and affect the soil productivity [4]. Soil salinization due to wastewater irrigation has been extensively reported during the recent years [57]. The major effect of wastewater on crop productivity is due to the presence of heavy metals, which are well-known to negatively affect crop productivity [58].

Wastewater may also carry viruses, bacteria, nematodes, and protozoa, which can cause different diseases [59]. Wastewater application may also affect the physic-chemical properties of soil, which, in turn, modifies the soil quality and fertility. Mireles et al. [60] reported that the agricultural sites (Mixquiahuala, Hidalgo and Tláhuac, D.F) previously irrigated with wastewater for more than 50 years in Mexico City show deterioration of the soil and now only certain plant species can be cultivated at these sites.

## 5. The Effect of Wastewater on the Physico-Chemical Properties of Soil

Wastewater application changes some physicochemical properties of the irrigated soil. Several previous studies have shown that the application of wastewater significantly changes the soil’s physical, chemical, and biological properties [80], which can, in turn, alter the biogeochemical behavior (mobility and bioavailability) of metals and other nutrients. Therefore, the variation in soil properties as a result of wastewater application can have a significant impact (both positive and negative) on the soil quality and crop productivity (Figure 1).

### 5.1. The Effect of Wastewater on Soil pH

The soil pH is the master variable that controls the partitioning of metals between the solid and solution phases of soil. The soil pH affects the adsorption/desorption of PTEs in soils [81], and, in turn, their biogeochemical behavior in the soil-plant system [82]. There exists a negative correlation between the soil pH and the PTE bioavailability to plants for several PTEs [83]. Most vegetables grow at their best in soils having a pH between 6.0 and 7.5 due to the increased availability of most of the nutrients. There exists a complex variation regarding the effect of wastewater irrigation on soil pH [84]. Wastewater is a source of acidic substances and irrigation using wastewater decreases the soil pH due to the decomposition of organic matter and the formation of organic acids in the soils [85,86]. In the majority of studies, the soil pH significantly increased after long-term irrigation with wastewater from different sources [87,88]. In some studies, however, soil pH was unaffected by long-term wastewater irrigation [89] while others reported decreased soil pH [19,89].

An increase in the soil pH as a result of the wastewater application might be due to the sulfate contents in the wastewater [90]. In general, the effect of wastewater irrigation on the soil pH depends on the pH of the wastewater source and the pH buffering capacity of the soil. Generally, the change in the equilibrium of the complex dynamic reactions taking place simultaneously in the soil may affect the soil pH after wastewater application. Previous studies [4,91,92,93] reported increased pH of soil under wastewater irrigation.

On the contrary, other studies reported decreases in the pH by wastewater irrigation [94,95,96,97,98]. The decrease in the soil pH after wastewater irrigation is due to the production of H^+^ ions produced during the oxidation reactions, while the decrease in soil pH is due to neutralization of the H^+^ ions by calcium carbonate in wastewater/sludge. Therefore, the overall effect of wastewater application on the soil pH depends on the initial soil pH as well as the cation/anion ratio and chemistry of the wastewater.

### 5.2. The Effect of Wastewater on Soil Organic Matter

Soil organic matter (SOM) is the second most important parameter determining soil quality, after pH. SOM governs the biogeochemical behavior (mobility/bioavailability) of metals and nutrients in the soil-plant system [81,99]. The processes controlling the metal behavior in soil mainly depend on the nature/type of organic matter. SOM is reported to exist in either a suspended or dissolved form in an aqueous medium [100]. When presented in the dissolved form, SOM can form metal-OM complexes with metals, while in the solid phase, SOM can adsorb metals from the aqueous medium [81]. Therefore, SOM can greatly affect (increase or decrease) the mobility of PTEs in soils and their availability to plants [101,102]. Organic matter (OM) also acts as a sink of essential nutrients which are important for plant growth [103]. The SOM content is also important for controlling the microbial activity in soil [104,105]. Large OM inputs enhance the microbial organism’s growth and block soil pores that cause decreases in soil infiltration and favor the anaerobic microbiological growth due to aeration problems in the soil [104,105,106,107].

Organic matter accumulation in the surface soil as a result of wastewater irrigation has been reported previously [4]. The application of wastewater will increase the OM contents in the soil which is considered a beneficial effect for soil [3]. The addition of organic matter into the soil through wastewater application improves the soil structure and the moisture content’s increased cation exchange capacity and helps to retain the metals and reduce their bioavailability and mobility and adds nutrients into the soils [108,109,110].

However, higher organic matter concentrations through wastewater application can have an adverse effect on the soil porosity and create anaerobic conditions in the root zone [111]. In addition, if an agricultural runoff having a higher OM concentration reaches the surface water, it may cause the dissolved oxygen depletion in the water, resulting in hypoxic conditions and thus, increasing the aquatic species mortality [108]. Continuous wastewater usage for irrigation significantly altered the water infiltration into soil due to the blockage of water transmission holes by organic matter [112]. The reduced soil water efficiency and lower water retention, as well as increased surface runoff and altered water infiltration, will have significant implications for the crop water use and the soil water balance.

### 5.3. The Effect of Wastewater on Soil Cations and Anions

Irrigation water comprises of inorganic constituents; primarily, the dissolved nutrients (Table 2) and salts, however, these salts vary greatly in both composition and concentration. The major constituents of dissolved salts are cations which include magnesium (Mg^2+^), sodium (Na^+^), and calcium (Ca^2+^) and anions which include sulfate (SO_4_^2−^), bicarbonate (HCO_3_^−^), and chloride (Cl^−^) [113]. Soil irrigation with wastewater generally adds significant quantities of cations, as well as their salts such as sulfates, phosphates, bicarbonates, and chlorides [113,114]. It is well known that the application of wastewater modifies the cation concentration in soil, which, in turns, affects the metal/nutrient balance between solid and aqueous phase soil [4,10]. However, the effect depends on the concentration of these cations in the applied wastewater.

Moreover, the concentration of these cations in the soil also varies with the type of vegetable cultivated because the nutrient uptake and accumulation varies with the vegetable type [4,115]. Vegetables are reported to be a rich source of nutrients and are capable of up-taking cations (Na, K, Ca, and Ba) in high quantities compared to crops and plants. Several studies [4,91] reported the increase in the concentrations of Ca, K, and Mg in the soil and in vegetables due to the wastewater application in irrigation.

In some studies, the application of wastewater provides the N, P, and K contents up to 4, 8, and 10 times more than needed by forage plants [116]. Thapliyal et al. [91] also reported total N contents values that are several times higher in soil irrigated with wastewater. Christen et al. [117] reported that long-term winery wastewater application on pastures resulted in the increased availability of K contents and had the potential of groundwater leaching and other water sources. Although the higher K ion content effects applied in the soil have not been studied extensively, its long-term application could cause the alteration of the soil physicochemical properties [118]. The excess concentration of Na is detrimental to the soil structure and reduces the soil’s capacity significantly to transmit water [4]. Wastewater application enhances the Cl concentration in the soil which exceeds the crop tolerance and the crops develop injury symptoms [119].

Overall, the worth of wastewater as a source of crop nutrients depends on the soil fertility level, the type and species of the crop grown, and the concentrations of the nutrients in the wastewater. The efficiency nutrient use for wastewater is nearly 100%. This is because the nutrients present in wastewater are commonly found in a dissolved form and, therefore, they are easily available for plant uptake. Moreover, the wastewater-induced nutrient supply matches the demand of crops because nutrients are supplied in patches with each irrigation, compared to synthetic fertilizers which are usually applied to crops in two to three splits [4,120].

The use of wastewater has been reported to save up to 94% and 45% of fertilizer required, respectively, for alfalfa and wheat [121]. It is reported that wastewater containing an average concentration of 35 mg/L of N, 10 mg/L of P, and 30 mg/L of K, largely fulfills the requirements of most vegetables and crops [6]. Usually, farmers benefit not only in terms of fertilizer savings but also in terms of improving the fertility of the soil. This also has an additional benefit for society by reducing the greenhouse gases produced during fertilizers, especially the nitrogenous manufacturing and supplying [122,123,124]. 

The effect of wastewater application on the soil nutrient status and the nutrient use efficiency is also reported in terms of crop production. It was observed that in comparison with the groundwater, the yield of marketable fruit was higher with wastewater [125,126]. Some other studies [4,127,128,129] also showed that wastewater irrigation significantly increased the dry and fresh forage yield of crops and vegetables in comparison with the well-water irrigation.

## 6. The Effect of Wastewater on Soil Microbial Community

The soil is a heterogeneous environment in both space and time and the microbial activity is focused at the localized sites on and around the organic residues. Decomposer communities undergo succession as inorganic and organic residues are changed. 

The application of wastewater has been reported to affect soil microbial activity and community establishment (Table 3). 

The effect of wastewater application on soil microbial activity can be direct or indirect via the changing of the soil physic-chemical properties [139,140]. In many low-income and developing countries, the use of wastewater for crop irrigation is one of several sources of pathogens [12]. Consequently, the quality of food and hygienic conditions remain critical even in areas where the irrigation water (wastewater) appears safe.

In metal contaminated soils, the change in the soil microbial diversity or shift from bacterial to fungal population has been reported [141]. The long-term municipal wastewater application has been revealed to reduce the diversity of the arbuscular mycorrhizal fungi [142] and some types of wastewater such as olive mill wastewater have been reported to have an impact on the microbial community structure [143,144]. Wastewater irrigation in soil altered the ammonia-oxidizing bacterial population and the *Nitrosomonas* and *Nitrosospira* species became dominant [144].

Wastewater use for irrigation may be the source of the beneficial bacteria for soils [145]. Wastewater and soil both have quite different characteristics, but are inhabited by a wide diversity of the bacteria. For example, in N cycling, the bacteria involved with the ability to remediate soil contaminants (for example, PTEs, antibiotics, or pesticides) may contribute to the improvement of the soil quality [146,147].

The increase in the activity of some enzymes (for example, laccases, hydrolytic, cellulases, phosphatase, proteolytic) has been reported in the soils irrigated with the treated wastewater [148,149,150,151,152]. This effect may be due to the provision of the organic carbon as suggested by the simultaneous increase in the activity of dehydrogenase, generally a parameter indicative of the biological oxidation of the organic compounds [152].

The irrigation of soil with wastewater is expected to stimulate different metabolic pathways and organisms through the supply of nutrients and organic matter. It is, thus, suggested that the irrigation of wastewater may stimulate microorganism activity involved in the biochemical balance of the elements such as N, P, and C [146,147]. However, the stimulation of the soil microbial activity and the abundance may have negative effects on the soil properties.

## 7. The Effect of Wastewater on Potentially Toxic Elements in the Soil-Plant System

### 7.1. The Effect of Wastewater on the Concentration of Potentially Toxic Elements in the Soil

Wastewater irrigation leads to the PTE accumulation in soil. In some countries, the groundwater contains high concentrations of PTEs [153,154,155,156,157,158], which also results in high levels of these elements in wastewater. Sewage water has been implicated as the potential source of PTEs such as Cd, Cu, Ni, Cr, Pb, and Zn in the soil, plants, and food items (Table 4). These PTEs have high environmental persistence due to their non-degradable nature and are readily accumulated in the soil to toxic levels [27,102,159,160]. Wastewater irrigation is well-reported to cause the disproportionate accretion of PTEs in soils [161,162]. A linear relationship of the wastewater irrigation period with the buildup of PTEs in the soil has been found [163,164]. As a matter of fact, the long-term soil irrigation with wastewater can be responsible for the soil contamination by PTEs [22,108,116].

Nowadays, the presence of PTEs in wastewater is abundant due to the excessive use of these elements in industrial activities and household articles [165,166,167,168]. Many studies worldwide have emphasized the risk of PTE accumulation in wastewater irrigated topsoil [169,170,171,172]. The levels of these PTEs in wastewater vary between regions and depend on the volume, source composition, and treatment of wastewater.

Several past studies from developing and developed countries reported PTE accumulations in the soil as a result of wastewater application. Compared to groundwater irrigated soils, high PTE contents in soils have been reported in different regions around the globe (Table 2) such as Fe, Cr, Co, Mn, Ni, Cu, Zn, and Pb in Mixquiahuala, Hidalgo, and Tláhuac, D.F. of Mexico City [60]; Cr, Pb, Ni, and Zn in Tongliao, China [186]; Cd, Cu, Zn, Pb, Cr, Mn, and Ni in suburban areas of Varanasi-India [187]; Cd, Cr, Cu, Pb, and Zn in the Bani–Malik wastewater treatment plant of Jeddah, Saudi-Arabia [132]; Cd, Cu, Cr, Ni, Pb, and Zn in Pakistan [188]; Cd, Ni, Cr, Zn, Cu, and Pb in Hanoi, Vietnam [189]; Zn, Cu, Mn, Cd, Pb, Ni, Fe, and Cr in Harare [190]; and Cd, Cu, Pb, and Zn near Nhue River, Vietnam [191]. Abdu et al. [176] reported soil concentrations of Pb (0.6–46 mg/kg), Cd (2.3–4.8 mg/kg), Ni (0–17 mg/kg), Cu (0.8–18 mg/kg), Zn (13–285 mg/kg), and Cr (1.8–72 mg/kg) in seven vegetable gardens from the three West African countries of Nigeria, Burkina, and Mali under 30 years of wastewater application. Khan et al. [76] reported increases in the PTE concentrations (Cr, Ni, Pb, Mn, and Cd) in soil irrigated with wastewater. Similarly, Khan et al. [20] reported a substantial buildup of Pb and other PTEs in the wastewater-irrigated soils compared to the control soil. Several other studies also reported PTE build-ups in the soil in different areas around the globe.

Despite the low levels of PTEs in most wastewaters, the soil may accumulate high levels of PTEs due to the continuous and long-term soil irrigation with untreated wastewater [4]. The long-term application of untreated and treated wastewater has resulted in significant increases of PTEs in the soil [19,92,142] as well as groundwater leachate through dumpsites [192].

Many studies conducted in Southeast Asian countries such as India, China, and Pakistan, where industrial effluent with sewage water (untreated or diluted) is widely used for irrigation found that Cd, followed by Pb, were the major metals which caused a risk to human health [4,21,188,193]. In most of these studies, the concentration of Pb and Cd exceeded the permissible limits for the PTEs in irrigation water; that is, the WHO/FAO standards of 5.0 and 0.01 mg/L for Pb and Cd, respectively [4,76,194]. Generally, due to higher mobility, Cd is a major relevant PTEs presenting a risk to human health; additionally, because it is bioavailable to plants at very lower concentrations that are not phototoxic but cause health risks to humans [167].

In peri-urban regions of Pakistan, vegetables and crops are frequently irrigated by wastewater without any primary treatment due to the non-availability of fresh water [195,196,197,198,199]. In different areas of Lahore-Pakistan, the continuous use of wastewater for irrigation in the agricultural areas has caused a buildup of highly toxic metals compared to the soil irrigated by groundwater [173]. Amin et al. [200] reported that the Pb concentration in the soil irrigated by wastewater was four times higher than the soil irrigated by tube-well water in Mardan-Pakistan.

Generally, irrigation with wastewater elevates the total and available PTE concentrations in soils. Heavy metals introduced into the soil via wastewater irrigation accumulate primarily in the surface layer and are generally more mobile and bioavailable than those released from the parent rocks [201]. Therefore, PTE addition to the soil by wastewater application may represent more threats to plant contamination than natural sources of PTE contaminations. The soil physico-chemical properties (electrical conductivity, pH, soil mineralogy, cation exchange capacity, and biological and microbial conditions) and the presence of soil organic and inorganic ligands greatly influence the mobile and bioavailable portion of PTEs in the soil [166,202]. In fact, all these soil properties and constituents control the basic physical, chemical, and biological processes that determine the fate and behavior of PTEs in soils [203,204].

### 7.2. The Effect of Wastewater on Potentially Toxic Element Accumulation in Plants

The soil is the direct pathway for the contamination of plants by PTEs via root uptake. Vegetables and crops irrigated by wastewater take up high concentrations of PTEs which may cause health risks to the users (Table 4 and Table 5). Several studies have demonstrated that wastewater irrigated plants may absorb and accumulate PTEs in concentrations greater than the maximum permissible limits (MPLs) with serious public health implications [4,73,164,205].

Several previous studies have also reported the high accumulation (above the toxic limit) of PTEs in different edible parts of crops/vegetables around the globe (Table 4): for example Pb, Cu, Zn, Ni, Cd, and Cr in *Beta vulgaris*, *Phaseolus vulgaris*, *Spinacea oleracea*, and *Brassica oleracea* [206]; Cr, Pb, Ni, and Zn in maize [186]; Cd, Cu, Cr, Ni, Pb, and Zn in vegetables [188]; Pb and Ni in Beta vulgaris [187]; Cd, Cr, Cu, Ni, Pb, and Zn in the vegetables [189]; and Zn, Cu, Mn, Cd, Pb, Ni, Fe, and Cr in *Zea mays* [190]. Kiziloglu et al. [207] reported that wastewater irrigation increased the Cu, Fe, Mn, Zn, Pb, Cd, and Ni contents of red cabbage and cauliflower plants. Similarly, the level of Cr, Pb, Ni, and Cd in the edible parts of okra were higher than the safe limit, with levels at 63%, 28%, 90%, and 83% in the samples, respectively [132]. They reported that the irrigation of okra with PTE enriched wastewater is not safe for human use. High concentrations of Cr, Cd, Co, Pb, Cu, Zn, and Ni were reported in spinach, cabbage, radish, and forage grasses when grown on sewage sludge-amended soils [208,209]. Similarly, the wastewater-induced increased accumulation of PTEs by vegetables than the allowable level by EU standards has been reported in Harare-Zimbabwe [205], Bejing-China [20], the industrial zone of Faisalabad-Pakistan [210], Varanasi-India [187], and Peshawar-Pakistan [70].

The soil-plant transfer of PTEs after the irrigation with wastewater depends on several factors relating to the soil, plant, and wastewater. Heavy metals may exist in soil in different forms such as free metal ion or complexed with various organic, inorganic, or soil constituents [204,211]. The soil-plant transfer of PTEs mainly depends on their chemical speciation [212,213,214]. Generally, the PTEs added to soil via wastewater application (anthropogenically) accumulate mainly in the topsoil and are usually have higher mobility and bioavailability compared to those deposited from their parent material [201,215].

The partition of PTEs in the soil and solid phases, as well as their soil-plant transfer after their introduction via wastewater irrigation, depend on soil’s physico-chemical properties (soil mineralogy, cation exchange capacity, pH, and microbial and biological conditions) and the presence of inorganic and organic ligands in the soil [18,27,216,217]. In fact, different soil physico-chemical properties control various soil physico-biochemical processes that govern the fate and behavior of the PTEs in the soils after being introduced by wastewater. For example, Mireles et al. [60] reported a low PTE accumulation in plants, probably due to the physico-chemical properties of the soils that prevent their translocation to plants in the agricultural soils of Mixquiahuala, Hidalgo, and Tláhuac irrigated with wastewater from Mexico City for more than 50 years.

Plant species have a diverse capacity for the accumulation and removal of PTEs from soil [218,219,220]. Certain plant species generally termed as hyper-accumulators can accumulate high levels of PTEs after wastewater irrigation [221,222,223,224]. Overall, hyper-accumulator plant species have the potential to accumulate PTE contents that are 100–1000 times higher compared to non-hyper-accumulating plants [204,218,225,226,227,228]. The edible parts of leafy vegetables grown under wastewater irrigation practice accumulate higher concentrations of PTEs than other vegetables [4]. Therefore, the soil-plant transfer of PTEs in wastewater irrigated soils also depends on the plant type being cultivated in that soil.

After metal uptake, the compartmentation of PTEs in different plant parts (root versus shoot or edible versus non-edible) also varies with the plant and the metal type. Generally, the majority of absorbed metals are stored in the plant root (>90%), with a small portion transferred to the plant shoot [166]. This sequestration of PTEs in the plant roots is due to the presence of endodermis or immobilization by negatively charged pectins within the cell wall (Pourrut et al., 2011). The heavy metal uptake and accumulation in different plant parts play an important role in their health effects [24,26,229,230,231,232]. Depending on the type of the edible part of the vegetable, the increased metal accumulation in the roots and shoots can be useful or toxic. For example, for leafy vegetables, the metal accumulation in the roots is useful, however, for tuber vegetables, a high translocation to its shoots is desired. The degree of the metal contamination also varies with the type of edible plant portion and its presence above or below ground. Generally, the risk of PTE contamination is higher for vegetables having consumable plant parts below the ground than those above the grounds.

Inside the plants, the compartmentation of PTEs in different plant parts is generally controlled by different transporter proteins [233,234,235]. Recent advancements in research at the cellular and genetic level have revealed numerous carrier proteins responsible for the root-shoot translocation of PTEs. These transporter proteins include HMA (heavy metal ATPase) [236,237,238], IRTP (iron-regulated transporter Proteins) [239], ZIP (zinc-regulated transporter Proteins) [240,241], CDF (cation diffusion facilitator) [242,243], and Nramp (natural resistance and macrophage protein) [244]. The expression of these metal carrier proteins is cell and metal specific and they may carry out different roles in different plant species.

Potentially toxic elements may accumulate at high levels in plants after wastewater irrigation. The excessive concentration of PTEs in plant tissue is capable of inducing various physiologically, morphologically, and biochemically toxic effects [18]. The heavy metals induce plant toxicity by disrupting the nutrient and water uptake and transport, altering the nitrogen metabolism, disrupting the activity of ATPase, reducing photosynthesis, interfering with plant growth, dysfunctioning the plant photosynthetic machinery in chloroplasts, and causing stomatal closure [245,246,247,248]. Heavy metals may also cause invisible symptoms of plant injury such as the browning of roots, necrosis, chlorosis, and leaf rolling [249,250,251]. At the cellular level, excessive PTE exposure can cause the enhanced production of reactive oxygen species (ROS), the alteration of cell cycles, and division and chromosomal aberrations [18,159,168,252]. Heavy metals have also been reported to causes protein oxidation, lipid peroxidation, and genotoxicity, most probably via ROS overproduction [216,253].

### 7.3. The Effect of Wastewater Irrigation on Food Chain Contamination and Human Health

Besides PTEs toxicity to plants, nowadays, food safety has become the most important public concern worldwide. The exposure of urban wastewater is multifaceted. Human health risks due to wastewater crop irrigation include the exposure of consumers and farmers to pathogens including the helminthes infections and inorganic and organic trace elements [4]. Direct exposure happens through the accidental inhalation, ingestion, or dermal contact in different ways: while using wastewater for domestic activities (for example, for dish cleaning or washing clothes), during working processes (for example, while managing the wastewater treatment and emptying the onsite sanitation facilities or reusing the wastewater for irrigation purposes), during flooding actions caused by heavy rains; and due to recreational activities (for example, bathing or swimming in lakes or rivers fed by the wastewater) [254,255,256,257].

Wastewater is discharged commonly into water bodies with little and no treatment due to the limited availability of treatment facilities in many low-income countries [4,10,12]. The release of untreated municipal and industrial wastewater into water bodies (oceans and seas) is a reason for the rapidly growing deoxygenated dead zones. It is estimated that wastewater disposal of water bodies affects about 245,000 km^2^ of marine ecosystems, as well as fisheries, livelihoods, and food chains [258].

Recent international data indicate that wastewater- and sanitation-related diseases are pervasive and growing alarmingly in countries where untreated wastewater is commonly used for crop irrigation. About 842,000 deaths in 2012 in middle- and low-income countries were linked with sanitation services, contaminated drinking water, and inadequate hand-washing facilities (WHO, 2014b). These diseases were mainly reported among children under 5 years of age [259,260].

Indirect exposure occurs through the use of contaminated drinking water or wastewater-fed fish and crops [261]. In the case of PTEs, humans can be exposed to these toxic compounds via several pathways such as dust inhalation, drinking contaminated water, or via atmospheric inhalation. However, the consumption of food contaminated with PTEs is considered to be the major pathway (>90%) of human exposure to PTEs [20,28,76,195]. Due to increasing the unchecked use of untreated wastewater for crop irrigation in many regions of the world, there is an increased risk of public exposure to the PTEs because of the consumption of food cultivated in sewage wastewater [21,60]. There are numerous studies in the literature supporting this assertion [4,20,21,28,76,186,188,195,196,255].

Clinical studies have revealed that serious systemic health issues can develop as a result of extreme dietary PTE accumulation and are linked with the etiology of a number of diseases, especially nervous system, cardiovascular, blood, and kidney, as well as the bone diseases [25,31,262]. The consumption of PTE contaminated vegetables can cause the depletion of nutrients in the human body that cause many problems in humans such as intrauterine growth retardation, disabilities with malnutrition, impaired psycho-social faculties, upper gastrointestinal cancer, and immunological defenses (Iyengar and Nair, 2000; Wang et al., 2012; Raja et al., 2015). These PTEs (for example, Pb and Cd) are capable of inducing carcinogenesis, teratogenesis, and mutagenesis; high Pb and Cd concentrations in edible plant parts were attributed to the occurrence of upper gastrointestinal cancer [29]. Moreover, Pb is also reported to cause improper hemoglobin synthesis, renal and tumor infection, elevated blood pressure, and the dysfunctioning of the reproductive system [166,245].

PTEs are even capable of inducing toxic effects to living organisms, including human beings, at very low levels due to the absence of proper defense mechanisms to mitigate the toxic effects of these metals and to remove them from the body. Therefore, much attention is given worldwide to food safety and risk assessment. Children and infants, in particular, are more vulnerable to wastewater contaminants [263] and their exposure to these contaminants was referenced in several articles [264].

Legislation regarding the use of wastewater for crop irrigation and associated health risks started in the early 19th century. During that period, wastewater use for crop irrigation in peri-urban fields induced catastrophic epidemics of numerous waterborne syndromes [265,266,267]. These health issues resulted in the establishment of some legislation at the national and international levels such as Great Britain’s Public Health Act, about the “discharge of rainwater in the river and of wastewater on the soil” [3,268].

In order to perform sanitary controls along the borders, the International Office of Public Hygiene was established [269]. The issue of wastewater-borne diseases also led to the development of underground sewage systems in many cities around the globe in the early 1950s [270]. Moreover, the international health and environmental/sanitary movement, generally backed and endorsed by European countries, resulted in a series of sanitary conferences/workshops/seminars on environmentally sustainable development.

Keeping in view the environmental and health risks associated with the use of wastewater disposal/use in the agricultural sector, WHO drafted the guidelines in 1973 on the “Reuse of effluents: methods of wastewater treatment and health safeguards”. These guidelines were later further updated in 1989 and 2006, keeping in view with epidemiological studies [38,271]. The parameters such as health risk assessments have now been included in these updated guidelines.

## 8. Health Risk Assessment after Food Chain Contamination by Wastewater Irrigation

Estimating the level of exposure of PTEs and tracing their routes of contamination to the target organisms are critical for understanding the health risks involved [25,272]. This is especially important in less developed countries, where the literacy and health risk awareness rates are very low. In low-income countries, many farm workers using wastewater for crop irrigation have been routinely exposed to poor hygiene conditions for most of their lives. Indeed, most of these farmers are either unaware of the risks or may accept these health risks for the benefits of their occupation with no other alternative/resources available as income.

Keeping in view the high volume use of untreated wastewater for crop irrigation and the low literacy rate in low-income countries, there exists a serious health risk of public exposure to PTEs due to the ingestion of vegetables/crops grown in wastewater (Table 5). Several literature studies, especially in low-income countries, support this assertion [21,28,76,195,273]. In order to trace the route and level of exposure to PTEs, a systematic risk assessment is necessary to avoid the possible health hazards and to make timely decisions/policies.

Nowadays, there is an increasing trend in estimating the health risk using soil contamination indices, soil-plant transfer factors, and metal contents in edible plant parts [25,31,274,275]. The most commonly used risk assessment parameters have been summarized in Table 6, which include the degree of contamination (Cdeg), the enrichment factor (EF), bioaccumulation potential (BAP), the uptake/transfer factor, translocation factor (TrF), hazard quotient (HQ), the health risk index (HRI), estimated daily intake (EDI), and lifetime cancer risk (ILTCR) [26,274,276].

## 9. Future Perspectives

The above-mentioned data show that wastewater crop irrigation has both positive and negative effects. However, by adopting and implementing some precautionary measures and practices, these negative effects of wastewater use can be minimized, making it a safe and reliable source of irrigation.

1. The environmental protection laws and their proper implementation totally differ in developing and developed countries. Generally, the cities in developed countries have well-established and adopted environmentally friendly practices and environmentally sustainable approaches regarding wastewater disposal, treatment, and reuse in the agricultural sector. However, the scenario is very much alarming in developing countries, especially in highly populated areas of the Indo-Pak Sub-continent. In future, more wastewater will be produced/disposed and more environmental and health risks will appear due to the rapid urbanization, industrialization, increase in the world’s population, food demand, economic development, and increase in living standards. Therefore, there will be a need for more systematic approaches in industrial and agricultural sectors to tackle this environmental and health dilemma. At the industrial level, the use of environmental-friendly processes and techniques with minimal use/production of waste material can be highly effective.
2. Similarly, the treatment of industrial wastewater before its discharge is also a key prerequisite to effectively alleviate its negative environmental effects. The proper establishment of wastewater treatment techniques can address the growing demands both in terms of quantity and quality. In the agricultural sector, the development of suitable irrigation approaches can be highly effective for its safe use. It is well-established that environmental contamination can be greatly controlled using a proper irrigation method. For example, drip irrigation has been regarded as the most environmentally friendly approach, which can mitigate up to 70% of environmental risks and leaching rates.
3. Climate change has recently emerged as a key environmental challenge. The uncertainty of this anthropogenic-assisted natural phenomena and irregularity of the environment has to be faced and tacked properly. The scattered pattern of droughts and rainfall over the temporal scale will aggravate water shortages in some areas while flooding other areas. Under such conditions, there will be a need for appropriate techniques and wastewater disposal infrastructure to collect, recycle, and distribute wastewater, protect the soil, and optimize the management.
4. In areas (arid, semi-arid) where fresh water supply is short, the mixing of wastewater with ground or surface water can greatly dilute the PTE concentrations in the applied (mixed) irrigation water. In this way, the risk of PTEs accumulating in soil and crops, as well as the associated health hazards, can be minimized. Similarly, the choice of vegetables/crops (low metal-accumulating species) cultivated using wastewater irrigation can also be a management strategy in areas where farmers have no choice but to use untreated wastewater for crop irrigation.
5. In order to effectively manage this environmental and health issue, there is need to properly implement laws and regulations on wastewater discharge and use in the agricultural sector, especially in less developed countries. The reports show that farmers in less developed countries do not pay enough attention to such laws and regulations, which results in environmental and health issue. Therefore, there is a need for strict regulatory systems, at the local, national, and international level, for effectively managing wastewater irrigation in the agricultural sector. Although abutment data are available regarding the wastewater use for crop irrigation, its effect (both positive and negative) on the soil, on plants, and on humans, there is limited data available with respect to the chemical speciation of the different contaminants (especially PTEs) in wastewater generated from different sectors at different time periods. Similarly, the plant physiological responses (overproduction of reactive oxygen species, lipid peroxidation) and tolerance mechanisms (activation of antioxidative enzymes, production of phytochelatins, glutathione, and so forth) remain unexplored under the wastewater crop irrigation scenario. It is possible that wastewater composition and chemical speciation of a contaminant may greatly vary in different municipal/industrial wastewaters during different seasons (summer and winter). Consequently, the environmental and health risk of that contaminant can vary under these circumstances. Further research work is needed in this regard.

## 10. Conclusions

Wastewater is used for crop irrigation as an alternative to freshwater. Wastewater collection, disposal, and use in agriculture have a long history. Nowadays, it has become a common practice in many countries around the globe to use wastewater for irrigation. Wastewater crop irrigation represents both opportunities and challenges with respect to its uses and its environmental/health effects. It appears beneficial in terms of a strategy to reuse and manage municipal/industrial water, conserve fertilizer and water, and to achieve certain environmental and social goals. This practice of wastewater crop irrigation has mitigated water deficit crises, to a large extent, especially in arid and semi-arid areas of the world. The nutritional value of wastewater has also been an attraction of its widespread use for crop irrigation. Simultaneously, untreated wastewater irrigation can provoke numerous environmental and human health issues. One of the main issues related to this practice is the build-up of heavy metals in soil, plants, food chains, and ultimately in human beings. 

When the environmental and human health issues related to wastewater crop irrigation are assessed globally, there exists a considerable difference between the developed and developing countries. The collection, recycling, and reuse of wastewater in the agricultural sector is better adapted and operated in developed countries compared to the developing world. Social, economic, corporate, and legislative issues are hindering its proper use in the developing world.

Keeping in view the rapid population growth and economic development as well as the uncertainty over climate change, the wastewater use in the agricultural sector may face many challenges. Therefore, the wastewater collection, recycling, and reuse have good prospects in the future, especially in rapidly growing and heavily populated cities, arid and semi-arid areas, and in developing countries. Thus, strategies and techniques for water saving should be methodized on priority. Finally, further scientific research regarding the use of wastewater irrigation is needed for the more effective and sustainable development and adaptation of wastewater irrigation systems, especially in less developed areas.

## Figures and Tables

**Table 1:** The wastewater production, collection, treatment, and reuse for crop irrigation in different countries in relation to the total agricultural area (Source, Aquastat-FAO) [9].

Country	Total Area (1000 ha)	Cultivated Area (1000 ha)	Total Cultivated Area (%)	Produced Municipal Wastewater (10^9^ m^3^/year)	Collected Municipal Wastewater (10^9^ m^3^/year)	Treated Municipal Wastewater (10^9^ m^3^/year)	Use of Treated Wastewater for Irrigation (10^9^ m^3^/year)
Australia	774,122	47,307	6.11	-	-	2	0.28
Brazil	851,577	86,589	10.1	-	-	3.1	0.008
China	960,001	122,524	12.7	48.51	31.14	42.37	1.26
Germany	35,738	12,074	33.7	-	5.287	5.213	5.183
India	328,726	169,360	51.5	-	-	4.416	-
Itlay	30,134	9121	30.2	3.926	-	3.902	0.087
Jordan	8932	322	3.60	-	0.115	0.113	0.103
Pakistan	79,610	31,252	39.2	3.06	-	-	-
South Africa	121,909	12,913	10.5	3.542	2.769	1.919	-
Turkey	78,535	23,944	30.4	4.297	-	3.483	-
UK	24,361	6279	25.7	4.089	4.048	4.048	-
USA	983,151	157,205	15.9	60.41	47.24	45.35	-
Canada	998,467	50,846	5.09	6.613	5.819	5.632	-
Sweden	44,742	2608	5.82	0.671	-	0.436	-

**Table 2:** The effect of wastewater and freshwater on vegetable nutrients and heavy metal contents.

Nutrients and Heavy Metal	Vegetables/Crops	Concentration in Vegetables Irrigated by Fresh Water (mg/kg)	Concentration in Vegetables Irrigated by Wastewater (mg/kg)	% Decrease or Increase	Reference
N	Lettuce	39,500	42,880	8.56	[61]
	Rice	296	453	53.04	[62]
	Coriander	402	499	24.13	
	Wheat	160	174	8.75	[63]
	Rice	135	142	5.185	
P	Lettuce	4480	5530	23.44	[61]
	Rice	28	38	35.71	[62]
	Alfalfa	0.26	0.27	3.85	[64]
	Rice	35	45	28.57	[65]
K	Rice	1364	835	−38.78	[66]
	Rice	225	312	38.67	[62]
	Coriander	416	517	24.28	
	Alfalfa	2.2	2.5	13.64	[64]
	Rice	106	230	116.98	[67]
Pb	Tomato	4.4	9.6	118.18	[68]
	Panicum	0.01	0.09	800	[69]
	Brinjal	4	14.15	253.75	[70]
	Radish	1	2.5	150	[71]
	Cypress	1.6	3.2	100	[72]
	Onion	11.2	2.7	415	[73]
	Garlic	8.15	4.94	165	
	Tomato	12.7	4.45	285	
	Brinjal	14.15	4.35	325	
Cd	Tomato	0.03	0.04	33.33	[68]
	Maize	0.02	0.03	50	[74]
	Cypress	0.05	0.06	20	[72]
	Radish	3.4	5.1	50	[75]
	Garlic	20	30	50	[76]
	vegetables, cereal crops	3.12	1.49	209	[21]
Ni	Tomato	4.67	8.33	78.37	[68]
	Cabbage	0.77	0.88	14.29	[77]
	Tomato	1.6	5.65	253	[73]
	Brinjal	3	7.45	148	
	Maize	0.62	1.12	80.65	[74]
	Lettuce	1.31	1.47	12.21	[78]
	vegetables, cereal crops	23.64	9.06	261	[21]
As	Maize	0.03	0.08	166.67	[74]
	Carrot	0.12	0.15	25	[78]
	Radish	0.49	0.5	2.04	[78]
	Radish	0.13	5	3746.15	[79]
Cr	Onion	5.05	1.05	481	[73]
	Garlic	2.6	1	260	
	Tomato	6.1	6.1	No	
	Brinjal	12.55	7.5	167	
	vegetables, cereal crops	19.2	9.07	212	[21]
Fe	Tomato	118	220	86.44	[68]
	Onion	6.15	26.15	325.20	[73]
	Brinjal	300	370	23.33	[62]
	Sunflower	140	324	131.43	[74]
	Lettuce	510	430	−15.69	[61]

**Table 3:** The number of microorganisms in soils colony-forming unit per gram (CFU/g) irrigated by wastewater.

Microbes	Microbes Count	Reference
Coliforms	3.3 × 10^2^ cfu/g	[130]
Coliforms Fecal Coliforms	4.39 × 10^3^/100 mL 7.5 × 10^7^ cfu/g	[131]
Fecal streptococci Fecal coliform	65 240	[132]
Bacteria (*Escherichia coli*, *Staphylococcus aureus*, *Streptococcus faecalis*)	7.6 × 10^7^ cfu/g 4.6 × 10^7^ cfu/g	[133]
Fungi (*Aspergillus niger*, *Aspergillus fumigatus*, *Aspergillus flavus*)	6.0 × 10^6^ cfu/g 9.0 × 10^6^ cfu/g	
Bacteria (*Lactobacillus plantarum*, *Pseudomonas aeruginosa*)	3.36 × 10^7^ cfu/g	[134]
Salmonella Shigella *Clostridium* bacteria	3.5 × 10^6^ cfu/g 5.4 × 10^4^ cfu/g 7.8 × 10^2^ cfu/g 5.1 × 10^4^ cfu/g	[135]
*Penicillium expansum* *Aspergillus* spp.	5.45 × 10^4^ cfu/g 1.30 ×10^5^ cfu/g	[135]
*Escherichia coli*	8.0 × 10^6^ cfu/g 3.8 × 10^6^ cfu/g	[136]
Bacteria *Actinomycetes* Fungi	1.34 × 10^7^ cfu/g 2.21 × 10^6^ cfu/g 9.99 × 10^3^ cfu/g	[137]
Total coliforms	2.1 × 10^3^ cfu/g 4.2 × 10^3^ cfu/g	[138]
Fecal coliforms	1.2 × 10^2^ cfu/g 4.2 × 10^2^ cfu/g	

**Table 4:** The heavy metal concentration in wastewater, soil, and plants in relation to the transfer and bioaccumulation factors.

Metal	Vegetable	Concentration in Wastewater (mg/L)	Concentration in Soil (mg/kg)	Concentration in Plant (mg/kg)	Transfer Factor	Bioaccumulation Factor	Reference
Cd	*Cupressus sempervirens*	0.06	0.03	0.06	0.5	2	[72]
Cd	*Raphanus sativus*	-	0.84	0.93	-	1.29	[20]
Cd	*Vicia faba*	-	0.11	0.1	-	0.9	[173]
Cd	*Oryza sativa*	0.01	3	1.1	300	0.4	[174]
Cd	*Spinacia oleracea*	10	5.8	15	0.6	2.6	[175]
Cd	*Lactuca sativa*	0.05	1	0.2	20	0.2	[176]
Pb	*Triticum*	-	41.56	2.77	-	0.1	[177]
Pb	*Raphanus sativus*	0.18	49.4	2.6	274.4	0.04	[20]
Pb	*Triticum*	0.585	411.7	26.23	703.8	0.064	[178]
Pb	*Convolvulus arvensis*	-	24.7	1.433	-	0.058	[179]
Pb	*Triticum*	0.1	33.4	2.3	334.0	0.069	[151]
Pb	*Oryza sativa*	-	5.1	0.37	-	0.073	[180]
Pb	*Cupressus sempervirens*	9.2	7.1	3.2	0.8	0.5	[72]
Zn	*Raphanus sativus*	-	157	57	-	0.41	[20]
Zn	*Daucus carota*	0.27	12.4	2.5	45.9	0.202	[181]
Zn	*Vicia faba*	0.36	0.42	0.07	1.2	0.2	[76]
Zn	*Amaranthus*	1	167	67	167.0	0.4	[176]
Zn	*Beta vulgaris*	0.24	1.7	25	7.1	14.7	[182]
Zn	*Hordeum vulgare*	0.19	1.4	32.2	7.4	23.0	[116]
Zn	*Citrus x sinensis*	0.02	134.22	4.15	6711.0	0.031	[89]
Ni	*Cupressus sepervirens*	7.1	11.3	4.7	1.6	0.4	[72]
Ni	*Oryza sativa*	1.03	35	1.8	34.0	0.051	[174]
Ni	*Raphanus sativus*	-	24.9	11	-	0.42	[20]
Ni	*Zea mays*	-	28.13	2.65	-	0.09	[183]
Ni	*Abelmoschus esculentus*	1.6	0.3	1.4	0.2	4.67	[132]
Ni	*Vicia faba*	0.04	0.55	0.09	13.8	0.2	[76]
Ni	*Triticum*	0.22	276.6	27.19	1257.3	0.098	[178]
Cu	*Cupressus sempervirens*	4.7	5.4	9.4	1.1	1.7	[72]
Cu	*Raphanus sativus*	0.2	5.4	1.2	27.0	0.222	[181]
Cu	*Raphanus sativus*	-	32.8	9	-	0.32	[20]
Cu	*Lactuca sativa*	-	7.4	8.05	-	1.088	[184]
Cu	*Xanthium strumarium*	0.616	0.768	0.791	1.2	1.0	[185]
Cu	*Vicia faba*	0.181	0.49	0.04	2.7	0.1	[76]
Cu	*Citrus x sinensis*	0.03	94.38	4.352	3146.0	0.046	[89]

TF = Transfer factor from wastewater to the soil, BF = Bioaccumulation Factor from soil to vegetable.

**Table 5:** The health risk assessment for vegetables cultivated using wastewater.

Metal	Vegetable	Estimated Daily Intake	Health Risk Index	Hazard Quotient	Reference
Cd	*Cupressus sempervirens*	0.00003	0.0315	0.000009	[72]
Cd	*Raphanus sativus*	0.00049	0.4879	0.000134	[20]
Cd	*Vicia faba*	0.00005	0.0525	0.000014	[20]
Cd	*Oryza sativa*	0.00058	0.5771	0.000158	[174]
Cd	*Spinach oleracea*	0.00787	7.8690	0.002156	[175]
Cd	*Lactuca sativa*	0.00011	0.1049	0.000029	[176]
Pb	*Triticum*	0.00145	0.4152	0.000398	[177]
Pb	*Raphanus sativus*	0.00136	0.3897	0.000374	[20]
Pb	*Triticum*	0.01380	3.9315	0.003770	[178]
Pb	*Convolvulus arvensis*	0.00075	0.2148	0.000206	[179]
Pb	*Triticum*	0.00121	0.3447	0.000331	[151]
Pb	*Oryza sativa*	0.00019	0.0555	0.000053	[180]
Pb	*Cupressus sempervirens*	0.00168	0.4796	0.000460	[72]
Zn	*Raphanus sativus*	0.02990	0.0997	0.008192	[20]
Zn	*Daucus carota*	0.00131	0.0044	0.000359	[181]
Zn	*Amaranthus*	0.03510	0.1172	0.009630	[176]
Zn	*Beta vulgaris*	0.01310	0.0437	0.003593	[182]
Zn	*Hordeum vulgare*	0.01690	0.0563	0.004628	[116]
Zn	*Citrus x sinensis*	0.00218	0.0073	0.000596	[89]
Ni	*Cupressus sempervirens*	0.00247	0.1233	0.000676	[72]
Ni	*Raphanus sativus*	0.00577	0.2885	0.001581	[20]
Ni	*Zea mays*	0.00139	0.0695	0.000381	[183]
Ni	*Abelmoschus esculentus*	0.00073	0.0367	0.000201	[132]
Ni	*Vicia faba*	0.00005	0.0024	0.000013	[76]
Ni	*Triticum*	0.01430	0.7132	0.003908	[178]
Cu	*Cupressus sempervirens*	0.00493	0.1233	0.001351	[72]
Cu	*Raphanus sativus*	0.00063	0.0157	0.000172	[181]
Cu	*Raphanus sativus*	0.00472	0.1180	0.001294	[20]
Cu	*Lactuca sativa*	0.00422	0.1056	0.001157	[184]
Cu	*Xanthium strumarium*	0.00042	0.0104	0.000114	[185]
Cu	*Vicia faba*	0.00002	0.0005	0.000006	[76]
Cu	*Citrus x sinensis*	0.00228	0.0571	0.000625	[89]

**Table 6:** The heavy metal soil contamination, soil-plant transfer, root-shoot translocation, and health risk assessment parameters used in different risk assessment and remediation studies.

Soil Contamination and Soil-Plant Transfer Indices	References	Risk Assessment Indices	References
Degree of contamination (Cdeg)	[277]	Root-shoot translocation factor (TrF)	[215]
Geo-accumulation index (Igeo)	[274]	Plant pollution index (PPI)	[274]
Contamination factor (CF), Enrichment factors (EF)	[274]	Estimated daily intake (EDI) or Average daily intake (ADI)	[31]
Mobilization factor (MF)	[215]	Hazard quotient (HQ)	[31]
Bioaccumulation factor (BF)	[278]	Maximum allowable daily intake (MDI)	[4]
Bioconcentration factor (BCF)	[215]	Life time cancer risk (ILTCR)	[31]
Plant enrichment factors (PEF)	[25,279]	Health risk index (HRI)	[4]
Soil-plant transfer factor (TF) or transfer coefficient (TC)	[274]	Tolerance index (TI)	[274]
